# Economic barriers, evidentiary gaps, and ethical conundrums: a qualitative study of physicians’ challenges recommending HPV vaccination to older gay, bisexual, and other men who have sex with men

**DOI:** 10.1186/s12939-019-1067-2

**Published:** 2019-10-17

**Authors:** Daniel Grace, Mark Gaspar, Ron Rosenes, Ramandip Grewal, Ann N. Burchell, Troy Grennan, Irving E. Salit

**Affiliations:** 1 Dalla Lana School of Public Health, University of Toronto, 155 College Street, Toronto, ON M5T 3M7 Canada; 2 Toronto General Hospital Research Institute, University Health Network, 200 Elizabeth Street, Toronto, Ontario M5G 2C4 Canada; 3 Department of Family and Community Medicine and MAP Centre for Urban Health Solutions, St. Michael’s Hospital, Unity Health Toronto, 30 Bond St, Toronto, ON M5B 1W8 Canada; 4 BC Centre for Disease Control, 655 West 12th Ave., Vancouver, BC V5Z 4R4 Canada

**Keywords:** Human papillomavirus (HPV), HPV vaccination, HIV, Gay, bisexual, and other men who have sex with men (GBM), Qualitative, Physician practices

## Abstract

**Background:**

The human papillomavirus (HPV) is the most common sexually transmitted infection (STI) worldwide. Gay, bisexual, and other men who have sex with men (GBM), and GBM living with HIV in particular, are disproportionately impacted by HPV-associated cancers. The HPV vaccine, given early enough in life, may markedly reduce the likelihood of such cancers. In Canada, most provincial insurance programs only cover HPV vaccination for GBM up to the age of 26. Our objective was to understand physicians’ everyday experiences and challenges in recommending HPV vaccination to older GBM patients.

**Methods:**

As part of the HPV Screening and Vaccine Evaluation (HPV-SAVE) Study, we conducted semi-structured interviews with 25 HIV-positive GBM patients who had received anal cancer screening and 15 service providers, including 13 physicians, who had arranged for anal cancer screening in the Canadian provinces of Ontario and British Columbia. In this analysis, we draw upon the 13 physician interviews, which were coded following Grounded Theory.

**Results:**

Physicians strongly supported the HPV vaccine for all GBM and considered it to be important for the management of HIV-related care. However, the overall support for HPV vaccination among physicians did not translate into consistent recommendation practices. There were two overarching factors that limited the strength/frequency of physicians’ vaccine recommendation practices. First, cost/insurance coverage for some older patients impacted if and how the HPV vaccine was discussed. Second, physicians had diverse perspectives on both the prevention and therapeutic benefits of vaccinating older GBM and the reality that national guidelines are incongruent with publicly funded vaccine programs for vaccinating patients over 26 years old. These two interrelated factors have co-produced an apparent *economic-evidentiary conundrum* for many physicians regarding how and for whom to offer HPV vaccination.

**Conclusion:**

Economic barriers coupled with evidentiary and guideline gaps have created clinical practice challenges for physicians and has resulted in different messages being communicated to some older GBM patients about how important HPV vaccination is for their health.

## Introduction

The human papillomavirus (HPV) is the most common sexually transmitted infection (STI) worldwide and is a cause for several types of cancers, including cervical, oral, oropharyngeal, penile, and anal cancers [1, 2]. Those living with HIV are at a significantly higher risk of developing HPV-associated cancers [3]. Notably, gay, bisexual, and other men who have sex with men (GBM) living with HIV (GBM-LHIV) are 100 times more likely to develop anal cancer than the general population [4]. Despite effective antiretroviral therapy, incidence rates of anal cancer among GBM-LHIV continue to rise internationally, making it one of the most important comorbidity threats to this population [5].

The HPV vaccine (HPV-4 or HPV-9) has demonstrated efficacy in preventing anal condyloma (i.e., warts) in males and anal dysplasia/anal intraepithelial neoplasia (i.e., pre-cancers that can develop into cancer) in GBM up to 26 years old [6]. In Canada, the HPV vaccine is currently only licensed for men 9 to 26.^1^ GBM older than 26 can receive primary prevention benefits from HPV vaccination as they may not yet have been exposed to all HPV types, including the oncogenic strains (HPV-16 and HPV-18), and their risk of infection or reinfection remains high [7]. Retrospective studies have also found that GBM who were vaccinated for HPV had a 50% reduction in recurrence of pre-cancer after treatment (i.e. removal of precancerous cells) than those who were not vaccinated [8].

Indeed, uncertainty exists in the scientific literature and policy over the benefits of vaccinating older adult males against HPV. While the prevention benefits of HPV vaccination for GBM who are 26 and under are consistently stated in policy reports, inconsistencies exist in the claims regarding the prevention and potential therapeutic benefits (i.e., helping to decrease the progression of pre-existing HPV infections into dysplasia or the progression of existing dysplasia into cancer) for older GBM. Some have described the “weak” recommendation strength for vaccinating HIV-positive patients and GBM older than 26, noting that the evidence to support HPV vaccination in these risk groups is “low” and “very low” respectively ([9] p8).

In Canada, the National Advisory Committee on Immunization (NACI) recommendations published in the Canadian Immunization Guide [10] state that “although there are no data on the efficacy of HPV4 or HPV9 vaccine in men 27 years and older who have sex with men, immunization with HPV4 or HPV9 vaccine should be strongly considered because of their increased risk of HPV related diseases.” A number of national and international policy recommendations [11–13] and “high risk” vaccination programs [14] have identified GBM and GBM-LHIV in particular, as priority populations for HPV vaccination. In the United Kingdom (UK), for example, the Joint Committee on Vaccination and Immunisation [12] has recommended that GBM and GBM-LHIV up to 45 years old should be vaccinated. As of April 2018, most regions in the UK offer free HPV vaccine to GBM 45 years old and younger [15].

Though these existing guidelines make recommendations about who should be vaccinated, in many contexts this guidance does not directly translate into what is actually made available via public programs. For example, in Canada, since 2016 most provincial insurance programs cover HPV vaccination for men who have sex with men only up to the age of 26 [16]. Otherwise, the vaccine cost ranges from $420 CAD (Gardasil-4) to over $560 CAD (Gardasil-9) for all three doses, which can be paid for with private health insurance for those who have adequate coverage, or out of pocket for those who are able to afford the costs [16].

Multiple studies have indicated that healthcare provider recommendations are one of the strongest factors that influence patients to be vaccinated against HPV [17–20] with one study demonstrating that GBM were 40% more likely to be vaccinated following a physician’s recommendation [19]. GBM often report limited awareness of HPV’s cancer risks, but do express a willingness to be vaccinated [21–24]. Side effects, stigma, and concerns over vaccine effectiveness have been documented as barriers to vaccination for GBM, though GBM-LHIV report higher levels of worry for HPV-associated illness [25, 26].

This body of research is primarily quantitative and has focused on the significance of provider recommendations for younger women and men. An exception to this is the recently published qualitative research from the HPV Screening and Vaccine Evaluation (HPV-SAVE) Study on the vaccine decision-making of older HIV-positive GBM patients in Toronto, Canada, who had received anal cancer screening [16]. Based on qualitative interviews with 25 HIV-positive GBM patients who had received anal cancer screening, Grace and colleagues explored the interrelated barriers to HPV vaccination including limited vaccine literacy and financial concerns [27]. Patients’ concern with HPV risk and anal cancer was highly structured by their physicians’ recommendations on these matters [28]. GBM-LHIV who said that the HPV vaccination was not strongly recommended to them by their doctor did not get vaccinated [16].

This qualitative analysis supports previous quantitative findings demonstrating a strong willingness among GBM to be screened and vaccinated for HPV related disease, with factors such a low income serving as significant barriers to vaccination [29]. However, to our knowledge, there is no qualitative study in Canada focusing on physicians’ viewpoints on HPV vaccination for older GBM-LHIV.

The objective of this analysis is to help account for physicians’ everyday experiences recommending the HPV vaccination to their patients in the Canadian context. Drawing on interview data with physicians, we explore how multiple factors can impact how doctors discuss HPV vaccination with their HIV-positive and HIV-negative GBM patients. The interviews reveal how physicians negotiated high vaccine cost and competing evidentiary claims about the prevention and therapeutic benefits of HPV vaccination for older GBM.

## Methods

Between November 2016 to October 2018, we recruited participants for in-depth qualitative interviews [30] about HPV and anal cancer screening. In addition to interviews with 25 GBM-LHIV patient-participants in Toronto, Canada, who had received anal cancer screening through HPV-SAVE, we interviewed 15 health service providers in Toronto, Ottawa and Vancouver, Canada. In this article, we focus on the data collected with providers to build upon recently published analyses of patients’ accounts of anal cancer screening [28] and HPV vaccination practices [16].

The sample of service providers was made up of 13 physicians and 2 clinical researchers who had experiences performing anal cytology screening (via a Papanicolaou or ‘Pap’ test) and/or referring patients to anal dysplasia clinics for screening or treatment of anal dysplasia [31]. A majority (*n* = 12) of the service providers were affiliated with the HPV-SAVE project, having administered anal Paps through the study. Below, we focus exclusively on the physicians’ accounts because the clinical researchers could not prescribe or administer the HPV vaccine. Of the physicians, 7 were HIV and infectious disease specialists, with 3 located in Vancouver, 1 in Ottawa and 3 in Toronto. Three of these were anal cancer screening specialists who are also investigators on the HPV-SAVE Study. The 6 remaining physicians were family doctors (general practitioners) who specialize in HIV-related care, sexual health, and/or who had a large GBM and HIV-positive clientele. Four of these family doctors practice medicine in Toronto and 2 practice in Vancouver.

Ethics approval was granted by the University of Toronto HIV Research Ethics Board. Participants were purposively selected by the research team and invited by email for the interview. We contacted a total of 19 providers for interviews of which 4 were unable to participate due to availability/scheduling issues. All participants provided written informed consent and agreed to the interviews being digitally audio recorded. Interviews were conducted in person (*n* = 12) in a private room in physicians’ clinics or offices, or by telephone (*n* = 3), and averaged 37 min in length (ranging between 25 min to over an hour). While some interviews were relatively brief, given the very specific focus of the interviews the data collected provided for in-depth and nuanced accounts of HPV vaccine recommendation decision-making. A community advisory board (CAB) was consulted throughout the process of data collection and analysis. The CAB included GBM-LHIV, community health advocates, social scientists, physicians, and epidemiologists.

A semi-structured interview guide was used to structure the conversation. The guide covered the following key domains: 1) *general description of provider’s clinical practice and/or research background*; 2) *knowledge and experience related to HPV, sexual health, and anal health*; 3) *knowledge and experience related to anal cancer screening practices (including anal Pap testing and anoscopy exams) and treatment*; and 4) *knowledge and experiences related to prescribing and administering the HPV vaccine*. While participants’ reflections throughout the full interview were taken into consideration—especially because the topic of the HPV vaccine came up routinely throughout the discussion—in the following analysis we draw extensively from the focused questions we asked regarding HPV vaccination.

The interviews were transcribed verbatim and analyzed in NVivo 11 software using a Grounded Theory approach [32, 33]. The interviews were analyzed in three steps. First, an open coding scheme was developed by organizing the data by all relevant ideas. Second, a focused coding technique was applied, which used the work in step one to build more substantial categories. And finally, a theoretical coding technique was used where the products of step two were brought together to create a consistent analytic thread.

## Results

The physicians we interviewed were strongly in favour of prescribing the HPV vaccine and believed it to be an important component of HIV-related care as well as for the health of all GBM regardless of their HIV serostatus. Physicians did not express any concerns regarding the safety of the HPV vaccine; a few indicated that the HPV vaccine had a similar side-effect profile to other vaccines and that they were “pro-vaccine”. As the HPV-9 vaccine was relatively new to the market at the time of the interview (and more expensive), physicians did not articulate any strong preferences for it above the HPV-4 vaccine.

Nonetheless, physicians varied in their opinions as to who should receive a strong or unequivocal recommendation for HPV vaccination. Some physicians were adamant that *all* their patients should be vaccinated for HPV. For example, one physician believed that HPV vaccination should be done “universally” regardless of a patient’s age so that “we can shut [anal cancer] clinics like this down in a decade” (General Practitioner 5). This participant argued that the slow response to offering HPV vaccine universally is directly responsible for an increase in HPV-associated cancers.

 Physicians said that they would clearly recommend the HPV vaccine to GBM who are age 26 and under, or those with health insurance. However, some physicians expressed that they recommended the vaccine to their HIV-positive patients regardless of age or whether or not they had insurance: “I’ve tried to talk about vaccination with everybody who’s [HIV] positive because of the greater rates of anal cancer in MSM who are HIV-positive” (General Practitioner 6). Another physician explained that aside from a select group of older GBM patients—“I mean, there is the 80-year-old guy I might not recommend it [to]”—they recommended HPV vaccination to most GBM patients and to all patients living with HIV (Specialist 8).

However, HPV vaccine recommendations for older men living with HIV was described as a “grey zone” (General Practitioner 12) with some practitioners communicating that they did not have specific strategies to ensure that everyone in their practice was vaccinated:

I think I do a good job of making sure patients are vaccinated against pneumonia, right? Because it’s now funded and I can get Prevnar and Pneumovax for my HIV-positive patients. But I haven’t necessarily been as good about counselling my HIV-positive patients about HPV vaccine, right? And I probably should’ve been doing that. (Specialist 1)

Below, we discuss the interrelated factors affecting the strength and frequency of vaccine recommendations for GBM: cost/coverage and the current state of evidence on HPV vaccination. After this review, we illustrate the relative complexity of the recommendation decision-making for physicians.

### Factors affecting universal and strong recommendations

Two interrelated factors were described as limiting the strength and/or frequency of the physicians’ recommendations for the HPV vaccine. The first was cost. Some physicians clarified that the only potential negative “side effect” of the vaccine was the financial burden: “But where is the medical harm? There isn’t any. [ …] In my mind, what’s the harm, aside from the $600?” (General Practitioner 4). Another physician declared: “There is not really any danger in getting the vaccine, but the disadvantages are, you know, several hundred dollars, unless you have coverage” (Specialist 8).

The second major factor affecting vaccine recommendation was a lack of clear evidence of the prevention and therapeutic benefits of vaccinating individuals over 26, including for GBM-LHIV, as well as unclear guidelines regarding this population’s vaccine needs. As one physician clarified:

The big unanswered question is the one that we grapple with all the time, is that is there a benefit of giving people who are sexually active, who’ve been sexually active, Gardasil now? Does that really lower their risk of getting an aggressive anal cancer, does it boost their immunity? That’s probably the biggest unanswered question, which is, in my practice, the most important question, because I am giving people Gardasil. But is it doing something? I don’t know. (General Practitioner 4)

Cost and insufficient evidence and national guidelines that are incongruent with publicly funded vaccine programs made it difficult for some physicians to determine *how* and *for whom* to offer strong and repeated recommendations for vaccination. Physicians described how the confluence of these issues produced an ethical conundrum where it was unclear whether or not the uncertain health benefits of vaccinating at an older age justified the personal expense to patients. For example, one physician explained that they would certainly recommend the HPV vaccine for anyone 9 to 26 years old but added that they did *not* systematically recommend it, and even somewhat discouraged it, for people outside of this age range:

And if they were over 26 and asking about it, I would say look, the evidence isn’t as strong because you’ve already been exposed at this point to so many [strains]. And it would be a sort of case-by-case discussion of well, the evidence isn’t as strong and at this point you’d have to pay for it, so this is what this would look like and I’m not routinely recommending it. (Specialist 1)

Thus some physicians’ decisions to recommend the HPV vaccine are based on *assumptions* of a patient’s presumed contact with HPV, which could range depending on their history of sexual behaviour.

The above physician also went on further to argue that the combination of the cost of the vaccine with the lack of compelling evidence created a challenging ethical dilemma: “It puts me in a – all of us as providers – I think in a bit of an ethical conundrum when we’re saying, I really think you need this thing, please give $300 to somebody else so that you can get it. I think that’s messy, right?” (Specialist 1). Similarly, another physician declared that it was problematic to offer people a medical suggestion that they may not be able to afford: “So that’s where I have problems when, you know, we are saying to people, well you actually will benefit with the vaccine, but you have to go buy it” (Specialist 9).

Conversely, one physician felt more certain about offering a strong recommendation for vaccination despite both the cost and unknown benefits of vaccinating older adults:

I would give it to everybody if we had easy access to it. Anyone who is above [26 years old] I would certainly let them know about it. I would offer it to them and if they were willing to pay for it, absolutely I would give it. I would be like anyone who’s had anal dysplasia or certainly anyone who’s had anal cancer or some kind of HPV related oral or other cancer, I would highly, highly recommend that they spend the money and get it. [ … ] I mean if they’ve got insurance, it’s a no brainer. If they don’t have insurance, I’m going to encourage it for everyone. But I would more strongly encourage it in somebody who has already had evidence of dysplasia or had some complication related to HPV. (General Practitioner 7)

While some physicians described feeling that the evidence on vaccinating older adults was more encouraging, others were less persuaded by the existing data. For example, one general practitioner articulated that there were clear benefits to being vaccinated at any age, but the cost considerations complicated recommendation practices:

A lot of people can’t afford [the vaccine] and it’s not covered and, you know, so we have to decide whether or not it’s really making a difference for them or would it just be completely sufficient for them to continue screening regular follow-up at the anal dysplasia clinic. I mean, then you miss the opportunity for the few patients who will develop an oropharyngeal cancer that there is a benefit there of having Gardasil. (General Practitioner 12)

One physician clarified that while the “HPV vaccine is amazing,” since most of the patients operating in this participant’s practice were HIV-positive they have already been infected with a “bad kind of HPV”, and the benefits of recommending vaccination were unclear. Although this physician argued that the vaccine may have “some benefit” for those with “pre-cancerous changes”, these clinical benefits were also read through a lens of pragmatism and cost considerations, with the physician opining: “Unfortunately, it’s a two-tier system. If they can afford it, or if they have insurance, they get the vaccine. If they can’t afford it and, you know, no insurance, most people don’t get the vaccine” (Specialist 13).

Similarly, a general practitioner argued that vaccinating people with insurance was a “no-brainer”, but that not vaccinating people with the financial resources was “unfortunately the reality of a lot of things in healthcare” (General Practitioner 12). Nonetheless, this participant argued that it was:

[Not] a great injustice that patients who don’t have coverage can’t get the vaccine, because I really am not convinced that it’s a huge difference in terms of prevention when you already have been exposed and you’re let’s say 60 years old and already have established risk factors. How would taking the Gardasil make a huge difference in your life compared to a 10-year-old boy who is, you know, before he starts his sexual life, you know, he is fully covered. (General Practitioner 12)

This participant discussed how his views on HPV vaccination recommendations were often contradicted by other providers who were more “enthusiastic” about recommending vaccination.

Physicians referenced existing guidelines and provincial vaccine insurance program for HPV vaccination. However, these did not always provide sufficient clarity on how to address the issue of vaccination for men older than 26. As one physician averred, “But even in that area, there is no clear guideline on who should be vaccinated and who shouldn’t” (General Practitioner 12). One specialist stated that while they agreed with the NACI guidelines and that HPV vaccination should be considered for all gay men, ultimately this was a decision they leave to their patients: “… I just put that to the patient and they can decide whether they want to spend the money or not” (Specialist 14).

One participant outlined the challenges of relying on existing recommendations, especially among their clientele that was mostly made of men with financial limitations. A host of factors was described as informing their decision to make a recommendation including age, level of HIV viral suppression, private insurance coverage, and a history of abnormal anal Pap results. However, if someone cannot afford the vaccine, the physician described that in such instances:

… it’s a weaker recommendation from me especially because my uninsured patients, many of them are unemployed and on social assistance and may not even have family members that can be a secondary source of funds for health stuff. So it’s a non-starter. If I know that someone does not have the ability to get the vaccine, we’re not going to have a very detailed discussion about it, which I think is different than another clinic. (General Practitioner 15).

Another physician questioned the logic of provincial health insurance program’s focus on men below the age of 26: “The issue is, you know, first of all, there is a cost thing, right? And some provinces have MSM programs, but a lot of them are capped at 26 again. 26 is interesting because it’s – that’s really based on the vaccine indication. So that’s a drug issue, right?” (Specialist 8). For this specialist, vaccine indication was understood to be a key driver of vaccine policy.

### Physician-patient discussions about HPV vaccination

A few physicians made it clear that they prioritized discussions on HPV and vaccination with their patients. As one HIV specialist declared: “So, I like, in my [practice] – I go over five things in every visit with every patient: anti-retroviral therapy, vaccines, and then HPV related disease is number three, and then other medical conditions and mental health and addictions” (Specialist 3). Other physicians we interviewed noted that it was often their patients who were initiating a discussion about HPV vaccination, with some commenting that it was about “fifty-fifty” between who raised the issue first (General Practitioner 2). Multiple physicians described that they had some patients who had heard about the vaccine from their friends and/or sexual partners and were bringing it up in clinic.

The cost of the HPV vaccine resulted in several physicians saying that they do not talk about HPV vaccination with certain patients, particularly with those patients they deemed to have financial constraints. One physician explained how economic factors entered into the recommendation decision-making and prevented discussions on vaccination with patients:

Yeah, and remember for a lot of my HIV patients, the majority live on ODSP [i.e. provincial disability insurance]. So, almost $600 is an insurmountable – I mean it’s just, you know, it’s just not feasible at all. So there’s really not a lot of point for me to bring that up to them and mention something I know they can’t afford. And I may be doing them a disservice because maybe they have resources I don’t know about or they have family members, but I think overall if I did that I’d probably cause more anxiety than good. (General Practitioner 2)

This physician went further to discuss how these complex conversations about money and strategies to access the vaccine could take up a lot of time during an already busy appointment, thus producing some hesitancy on their part to bring up the vaccine in the first place:

The problem with HPV is that it’s not good enough just to not see it there [on their medical charts]. Then you’re like, okay, does the person have coverage, do they have money? So then it becomes this, so it’s no longer a routine thing. With HPV, I have to have the discussion first and then they come in and we get talking about something else and the discussion may not happen, whereas if it was covered for everybody, it would just be one of those things that that’s done automatically. (General Practitioner 2)

To mitigate these barriers of cost, some physicians offered different access strategies for their patients, including only taking two doses of the vaccine instead of three,^2^ and directing patients to clinics where they know they can get the vaccine more inexpensively. These examples help to illuminate the navigation work some physicians have taken on in order to help get their income vulnerable patients access to the HPV vaccine.

## Discussion

Our interviews with physicians revealed how they frequently faced a complex *economic-evidentiary conundrum* regarding how and for whom to offer HPV vaccination. This ethical conundrum was a product of two intersecting and mutually reinforcing factors: *high cost* and *limited evidence*. In general, physicians said that they strongly supported the HPV vaccine and considered it to be an important biomedical intervention for the comprehensive care of those living with HIV as well as for the general health of GBM who were HIV-negative. However, the overall support for HPV vaccination among physicians did not translate into providing consistent recommendations to all patients [37]. As such, this *economic-evidentiary conundrum* made it difficult to establish equitable and standard recommendation practices across the board for patients and created additional labour for physicians in terms of selecting when and how to bring up recommendation and counselling patients without insurance about the advantages and disadvantages of HPV vaccination. Put differently, a complex dynamic between the interrelated factors of cost and evidence for clinical efficacy among older GBM patients structured physicians’ stratified recommendations in their practices.

First, and perhaps not surprisingly, cost/insurance coverage concerns for some patients in their practice impacted if and how HPV was discussed [38, 39]. Limited research has quantitatively explored how higher socio-economic status (SES) is positively associated with an increased willingness to be vaccinated for HPV [40, 41]. As such, our analysis raises questions concerning the need to examine the role of SES in vaccine decision-making—and vaccine recommendation practices—particularly for older GBM. Second, physicians had diverse perspectives on the absence of strong evidence for the prevention and therapeutic benefits of vaccinating older individuals and how this relates to a lack of clear guidelines for vaccinating patients over 26 years old.

These two interrelated factors co-produced an ethical conundrum for many physicians regarding how and for whom to offer strong and repeated recommendations for HPV vaccination. In the absence of a patient having cost/coverage barriers, any ambiguities in the current state of the evidence regarding the prevention benefits of vaccination at an older age appeared to be a negligible concern for many physicians since there would be, at a minimum, no harm done in vaccinating patients. Furthermore, there could be potential benefits in preventing infection by some oncogenic strains which have not yet been contracted; or there may be benefit in reducing the progression or recurrence of anal dysplasia among those already infected with oncogenic HPV.

Our analysis builds on previously published work on the HPV vaccine decision-making of older GBM-LHIV in Ontario. We found that receiving a strong recommendation by their physician was a primary facilitator to be vaccinated. Based on these interviews we argued that we have observed a form of “structurally produced vaccine hesitancies” explaining that:

Rather than a property of individuals refusing vaccination, conceptualizing the production of vaccine hesitancy in this way elucidates how social systems—i.e. media, health promotion campaigns, public policy, physician recommendations—serve to create environments and knowledge systems in which people are less likely to become vaccinated due to dominant discursive frames of risk and persistent resource barriers to biomedical interventions ([16] p10).

The interviews with physicians presented in this paper critically build upon this previous work and helps to illuminate the gaps in evidence and economic conditions – principally the high market price of the HPV vaccine matched with a lack of public programming to fund vaccination for those older adults without private insurance – that have led some physicians to be hesitant or uncertain about recommending HPV vaccination to their older GBM patients. In quantitative research with 1531 unvaccinated men attending HIV care in Ontario, only 18% agreed with the statement: “my doctor thinks I should get the HPV vaccine” [42].

Physicians described the significant value of generating increased “real world” evidence and clear guidelines surrounding the secondary prevention benefits of HPV vaccination for older patients, including diverse GBM, noting that it would be easier to communicate the importance of HPV vaccination even when cost remains a barrier. Such evidence may help to revolve recent critiques of the “weak” evidence for vaccinating GBM and HIV-positive persons ≥26 years old [9].

Vaccinations remain one of the most important public health strategies for preventing infectious diseases. Disparities in HPV vaccination access among GBM-LHIV raise important questions of health inequity because these differences are “systematically associated with social disadvantage in a way that puts an already disadvantaged social group at further disadvantage” ([43] p256). Physicians working with clients living with HIV are confronted with the challenges of reconciling vaccine access inequities in their everyday practice. While we do not fully know the protection benefits of the HPV vaccine for some older patients, we do know that under the current ‘patchwork’ system of drug coverage in Canada (which lacks a national pharmacare program) [44, 45], these potential significant cancer-preventing benefits of the vaccine are largely reserved for only a select subgroup of older GBM patients who can afford vaccination. In short, persistent inequities—and various tiers of vaccination access—remain even in Canada’s so-called ‘universal’ public healthcare system [46, 47]. The current lack of affordability of the HPV vaccine limits access for some economically disadvantaged groups and makes it more challenging for physicians to establish equitable practices in their clinics. Beyond public health messages of improving patient health literacy and models of provider-patient communication, we argue that it is necessary to consider the upstream determinants of vaccination access in Canada [48].

### Limitations

Our study was limited by speaking to physicians whose clienteles are made up predominately of HIV-positive patients and/or GBM. These physicians were also highly networked with anal dysplasia clinics in their cities, or they were themselves anal dysplasia specialists. Thus this group most likely had a higher degree of knowledge surrounding HPV research and recommendation practices compared to the majority of other physicians in Canada. While some non-specialist physicians may not experience the same ethical conundrums expressed here, we believe that the findings presented in this paper speak to larger structural issues of relevance to all physicians in Canada: how economic constraints and evidentiary gaps serve to structure their work and produce differential access to healthcare for their patients.

## Conclusion

Cost barriers, coupled with the current evidence base of the benefits for older GBM, produced an ethical conundrum for physicians when making HPV vaccination recommendations. The desire to provide equitable access to healthcare was thwarted by significant price barriers for older GBM and persistent evidentiary gaps. We echo calls from the physicians we interviewed for the necessity of increasing research efforts—or “real world” evidence—with older patients to better understand the benefits of HPV vaccination for diverse people, including HIV-negative and HIV-positive GBM. Our analysis also raises significant health equity concerns regarding some older GBM being put potentially at risk for HPV vaccine-preventable cancers because of, at least in part, the prohibitive cost of the vaccination.

## Data Availability

The full qualitative transcripts for this study are not publicly available for reasons of research ethics and participant confidentiality.

## Abbreviations

CAB: Community advisory board
GBM: Gay, bisexual, and other men who have sex with men
GBM-LHIV: Gay, bisexual, and other men who have sex with men – Living with human immunodeficiency virus
HIV: Human immunodeficiency virus
HPV: Human papillomavirus
HPV-SAVE Study: Human papillomavirus - Screening and Vaccine Evaluation Study
ODSP: Ontario disability support program
SES: Socio-economic status
STI: Sexually transmitted infection

## Footnotes

1 In the United States, the indications for HPV vaccine have recently changed on the Gardasil product monograph to the upper age limit of 45 for males. We suspect that such changes will soon occur to the Canadian monograph, which will likely impact licensing.

2 One participant referenced the WHO recommendations on vaccine dosing to account for this practice [34–36].
